# Elucidating the Calcium-Binding Site, Absorption Activities, and Thermal Stability of Egg White Peptide–Calcium Chelate

**DOI:** 10.3390/foods10112565

**Published:** 2021-10-25

**Authors:** Zhijie Bao, Penglin Zhang, Na Sun, Songyi Lin

**Affiliations:** National Engineering Research Center of Seafood, School of Food Science and Technology, Dalian Polytechnic University, Dalian 116034, China; zhijie_bao@163.com (Z.B.); Zhangpl134@163.com (P.Z.); sunna@dlpu.edu.cn (N.S.)

**Keywords:** egg white, peptide–calcium chelate, characterization, thermal stability, calcium supplement

## Abstract

With the current study, we aimed to determine the characteristics and calcium absorption capacity of egg white peptide–calcium complex (EWP-Ca) and determine the effect of sterilization on EWP-Ca to study the possibility of EWP-Ca as a new potential calcium supplement. The results of SEM and EDS showed a high calcium chelating ability between EWP and calcium, and the structure of EWP-Ca was clustered spherical particles due its combination with calcium. The FTIR and Raman spectrum results showed that EWP could chelate with calcium by carboxyl, phosphate, and amino groups, and peptide bonds may also participate in peptide–calcium binding. Moreover, the calcium absorption of EWP-Ca measured by the intestinal everted sac model in rats was 32.38 ± 6.83 μg/mL, significantly higher than the sample with CaCl_2,_ and the mixture of EWP and Ca (*p <* 0.05) revealed appropriate calcium absorption capacity. The fluorescence spectra and CD spectra showed that sterilization caused a decrease in the content of α-helix and β-sheet and a significant increase in β-turn (*p <* 0.05). Sterilization changed the EWP-Ca structure and decreased its stability; the calcium-binding capacity of EWP-Ca after sterilization was decreased to 41.19% (*p <* 0.05). Overall, these findings showed that EWP could bind with calcium, form a peptide–calcium chelate, and serve as novel carriers for calcium supplements.

## 1. Introduction

Eggs are rich, high-quality protein foods essential for daily living [1]. Due to the traditional dietary habits of Chinese residents and the specific needs of some industries, there is a significant demand for egg yolks. In many production processes, egg white resources, representing a good source of peptides, are wasted [2]. Peptides have several beneficial effects on bodily functions, such as hydrolysates of proteins [3]. Due to the unique characteristics of peptides, such as low molecular weight, multi-activity, and easy absorption [4], there has been an increase in the research of the bioactive properties of egg white peptide (EWP) obtained by enzymatic hydrolysis of egg white protein, such as antihypertensive [5], anti-fatigue [6], anti-oxidation [7], and anti-inflammatory effects [8,9].

In the human body, calcium is one of the richest inorganic elements. It accounts for 1.5–2.2% of the human body weight [10] and is involved in the physiological processes of bone development as an essential mineral [11]. Multiple researchers have reported that calcium is an essential element for fracture prevention and bone density. The deficiency of calcium could cause a series of diseases, such as rickets, osteoporosis, and hypertension. However, calcium deficiency in the human body is still a general phenomenon worldwide, particularly among older women [10,11,12]. According to reports, the absorption rate of calcium in the human body is mainly determined by the soluble calcium content in the duodenum and proximal jejunum [13]. However, calcium ions and antagonists such as oxalate, phytate, and cellulose can form precipitates in the intestines, causing the solubility of calcium to decrease [14,15]. Thus, multiple kinds of calcium-containing complexes were evaluated to determine the ability to treat and avoid calcium deficiency, such as calcium gluconate, calcium lactate, and calcium carbonate. However, these calcium supplements showed a poor effect [16]. Recently, there has been an increase in the study of calcium supplements from food resources, particularly peptide–calcium chelate. It was found that these calcium supplements could reduce the formation of insoluble calcium compounds and promote calcium absorption [17]. Currently, various kinds of food source calcium-binding peptides have been reported, such as wheat germ peptide–calcium complex [18], cucumber seed peptide–calcium chelate [12], pig bone collagen peptide–calcium chelate [19], and chicken foot broth byproduct peptide–calcium chelate, among others [20].

The purpose of this research was to prepare an EWP-calcium complex (EWP-Ca) and investigate its characterizations, ability to promote calcium absorption, and stability during the sterilization process. We anticipate that these findings will provide a theoretical basis for the potential use of EWP-Ca as a new type of calcium supplement.

## 2. Materials and Methods

### 2.1. Materials

Egg white powders were obtained from Heilongjiang ZhongNongXingHe Biotechnology Co., Ltd. (Jiamusi, China). The chemicals in our experiment were of analytical grade.

### 2.2. Preparation of Egg White Peptide and Egg White Peptide–Calcium Complex

The preparation of egg white peptide (EWP) was performed as described by Lin et al. [21] with some modifications. We dissolved the egg white powder (5% *w*/*v*) and heated at 90 °C for 30 min, then hydrolyzed the solution by Alcalase (3% *w*/*v*) at 50 °C for 3 h at pH 10.66. After that, the enzyme in the solution was inactivated for 10 min through a boiling water bath, followed by centrifugation (12,000*× g*, 4 °C, 10 min), and the supernatant was collected for freeze-drying and obtaining the EWP powders.

The EWP-calcium complex preparation was carried out following Sun et al. [15]. First, a solution with 50 mg/mL final content of dissolved EWP powders in ultrapure water was prepared. After that, we added CaCl_2_ to the solution and made the mass ratio of EWP to calcium at 3:1 *w*/*w*. This solution was mixed by stirring at 50 °C and pH 8 for 60 min, and then ethanol (85%) was added to remove the unbound calcium. Finally, the solution was centrifuged (12,000*× g*, 4 °C, 5 min). We collected the supernatant for freeze-drying and the obtained product was named the EWP-calcium complex (EWP-Ca).

### 2.3. Scanning Electron Microscope Analysis and Energy-Dispersive X-ray Spectroscope Analysis

Scanning electron microscope analysis was performed using the previously described method [22]. The samples were coated with gold on a bronze stub. The samples’ microcosmic morphology was measured using scanning electron microscopy (SU8010; Hitachi, Tokyo, Japan) with an accelerating voltage of 5 kV. Energy-dispersive X-ray spectroscope (EDS) analysis of EWP and EWP-Ca was used as described previously [23].

### 2.4. Fourier Transform Infrared Spectroscopy

Fourier transform infrared (FTIR) spectra of samples were evaluated using an FTIR spectrometer (PerkinElmer, Salem, MA, USA) as described previously [24].

### 2.5. Raman Spectrum Analysis

The Raman spectra of EWP and EWP-Ca were evaluated using the Horiba LabRAM HR Evolution micro-spectrometer (Horiba Jobin Yvon, France) as described previously [25].

### 2.6. Promote Calcium Absorption Analysis

The intestinal everted sac model in rats was set up following Hou et al. [26]. Wistar male rats in the bodyweight range of 180–220 g were fasted for 12–16 h in laboratory condition with free access to water before sacrifice. We collected 7 cm of the small intestine immediately and rinsed the intestinal contents in a 4 °C standard saline solution. One end of the small intestine was ligatured under continuous oxygen (95% O_2_) in the buffer at 4℃. The buffer was a mixed solution of 136 mmol/L NaCl, 8.17 mmol/L KCl, 1.0 mmol/L MgCl_2_, 11.1 mmol/L glucose, and 20 mmol/L HEPES. Finally, the intestine was turned over and the buffer solution was injected into the drug-free, ligation end of the small intestine.

Anhydrous calcium chloride (CaCl_2_), casein phosphor peptide calcium (CPP–Ca), and a mixture of EWP and Ca (EWP + Ca) were used as control groups. The CaCl_2_, CPP-Ca, EWP + Ca, and EWP-Ca were dissolved in the buffer at 25 °C and we kept the calcium content at 2 mmol/L. We immersed the intestinal everted sac into the sample buffers and incubated them under the condition of continuous oxygen for 2 h. Then, the solution in the intestinal sac was collected, and the calcium content was evaluated by the atomic absorption method.

### 2.7. Nano LC-ESI-MS/MS Analysis

The composition of EWP-Ca was analyzed by Nano LC-ESI-MS/MS as described by Wang [27]. Sample separation and fragmentation were executed using a Thermo Scientific EASY-nLC 1000 System (Thermo Fisher Scientific, Waltham, MA, USA) coupled to a Triple TOF 5600 mass spectrometer (AB SCIEX, Redwood City, CA, USA). The elution was performed by a gradient of 1–90% mobile phase B (A phase: 0.1% aqueous solution; B phase: ACN solution containing 0.1% manic acid) at a flow rate of 400 nL/min over 60 min by Acclaim PepMap C18, 75 μm × 25 cm chromatography column. The mass spectra were extracted and annotated with amino acid sequences using the Paragon™ Algorithm: 5.0.0.0 (revision number: 4767, SCIEX, USA) to extract the mass spectra and annotate with amino acid sequences. The peptide identification was conducted using ProteinPilot^TM^ 5.0 software (revision number: 4769, SCIEX, USA).

### 2.8. Analysis of Stability of EWP-Ca during the Sterilization Process

The sterilization conditions for this experiment were determined under 85 °C for 10 s, following standard market EWP production procedures. Unsterilized EWP-Ca was the control group.

#### 2.8.1. Fluorescence Spectra

Fluorescence spectra were evaluated by the Hitachi F-2700 fluorescence spectrometers (Hitachi High-Tech Group, Hitachi, Japan). We prepared the samples (0.1 mg/mL) by deionized water and measured the following parameters: excitation wavelength, 295 nm; scanning range, 295–330 nm; and slit width, 5 nm, for both excitation and emission.

#### 2.8.2. Circular Dichroism Spectra

Circular dichroism (CD) spectra were evaluated by Jasco J-1500 spectropolarimeter (Jasco Co., Tokyo, Japan) as described by Bao et al. [28]. Samples (0.1 mg/mL, pH 7.4) were prepared and measured with the following parameters: scanning range, 190–260 nm; scanning speed, 100 nm/min; bandwidth, 2.0 nm; data interval, 1 nm.

#### 2.8.3. Calcium-Binding Capacity Analysis

After sterilization, absolute ethanol was added to the EWP-Ca solution (10 mL) to remove the unbound calcium, making the final concentration 85%. The solution was centrifuged (12,000*× g*, 4 °C, 5 min) and pellets were collected and dried in a vacuum freeze-dryer to determine the calcium content by atomic absorption method and calculate the calcium-binding capacity.

### 2.9. Statistical Analysis

All experiments were carried out in triplicate, and data are expressed as mean ± standard. All data were imported into Statistix 8.1 software package (Analytical Software, St Paul, MN, USA), and the statistical significance was set at *p* < 0.05.

## 3. Results

### 3.1. SEM and EDS Analysis

**Figure 1:**
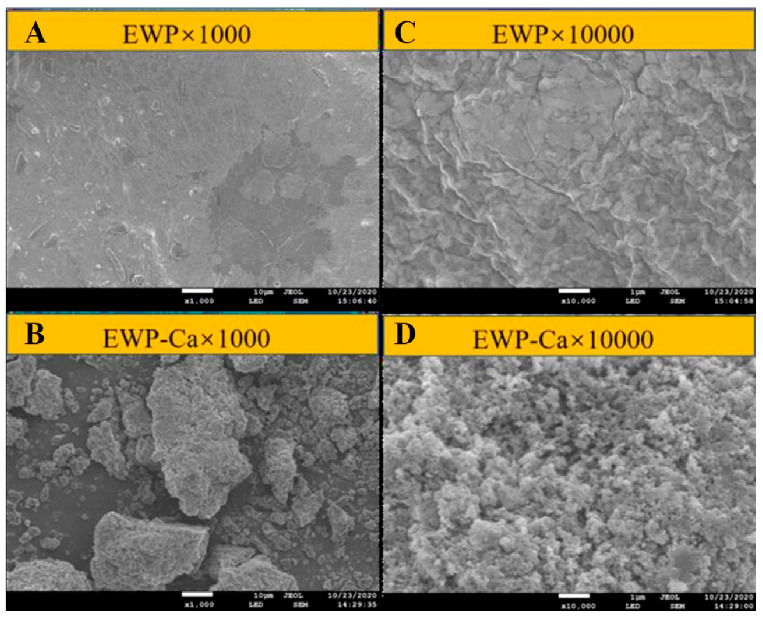
SEM images of EWP and EWP-Ca. (**A**) SEM image of EWP at a magnification factor of 1000×; (**B**) SEM images of EWP-Ca at a magnification factor of 1000×; (**C**) SEM images of EWP at a magnification factor of 10,000×; (**D**) SEM images of EWP-Ca at a magnification factor of 10,000×.

**Figure 2:**
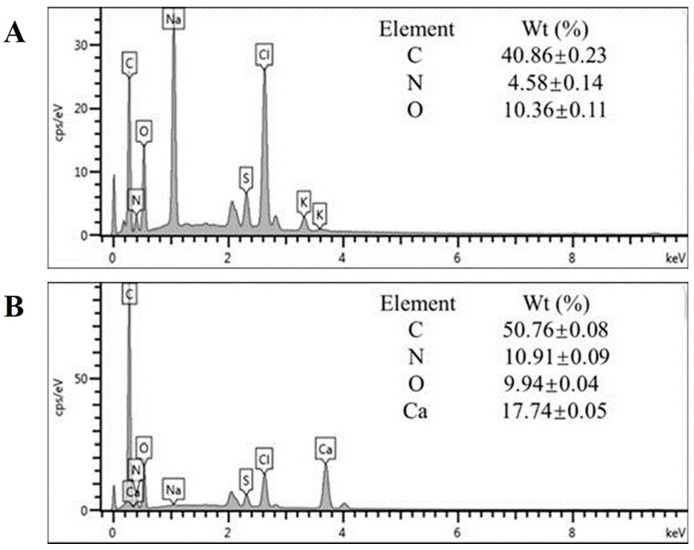
The EDS pattern and percentage of EWP (**A**) and EWP-Ca (**B**) elements.

The *Microstructures* of EWP and EWP-Ca are displayed in Figure 1 and Figure 2. SEM was used to observe the microstructure under the 1000× and 10,000× field of view. As shown in Figure 1A, the surface of EWP under the field of view of 1000× presents a smooth flake structure, while the surface of EWP-Ca shown in Figure 1B presents many smaller irregular shapes, and some small protrusions are gathered. While under the field of view of 10,000×, it was observed that the surface of EWP is uneven (Figure 1C). In comparison, the surface of EWP-Ca had clustered spherical particles (Figure 1D). In addition, many white spots could be found on the surface of EWP-Ca. The main reason for the difference between EWP and EWP-Ca may be that the coordination bond between peptide and the calcium caused the internal structure to change, leading to a compact structure of EWP-Ca [29]. The phenomenon found in our study is in line with Zhang et al. [30], who reported that after being chelated with zinc, oyster protein hydrolysates–zinc complexes formed more compact particles, which indicated that the metal ions could promote aggregation. Our findings are also in agreement with research on chicken foot broth byproduct peptides–calcium chelate, which reported that chelate with calcium ions resulted in a more compact, granular, and crystal-like structure [20].

The EDS analysis is usually used to analyze the chemical composition of samples [31]. As shown in Figure 2A, the result of EDS analysis indicated that EWP contained elements C, N, and O, and the contents of three elements were 40.86%, 4.58%, and 10.36%, respectively. The result of EWP-Ca is shown in Figure 2B; the peak of element Ca was found, and the content was 17.74%. Therefore, it could be inferred that the EWP was chelated with calcium. In addition, the result of EDS also showed peaks for sodium, sulfur, and chloride, presumably introduced by other reagents added during the experiment. Based on the above findings, the SEM results indicate that EWP-Ca has clustered spherical particles. Meanwhile, the EDS analysis demonstrates a high calcium chelating ability between EWP and the calcium ions.

### 3.2. Fourier Transform Infrared (FTIR) Spectroscopy Measurement

The formation and composition of organic functional groups could be measured by FTIR spectroscopy, such as C-O, N-H, and O-H [32]. It is well known that FTIR spectroscopy has two key regions: one of them is the functional group region, which is between the range of 4000*–*1300 cm^−^^1^, used to evaluate the types and changes of groups, and the other one is the fingerprint region in the range of 1300*–*600 cm^−^^1^, used to indicate molecular characteristics and structure [20]. Furthermore, the amide-I-vibration at 1700*–*1600 cm^−^^1^ and the amide-II-vibration at 1600*–*1500 cm^−^^1^ were also two crucial amide vibration modes caused by the stretching and the deformation of the C-O, N-H, and C-N [33]. Figure 2 shows the FTIR spectroscopy of EWP and EWP-Ca.

**Figure 3:**
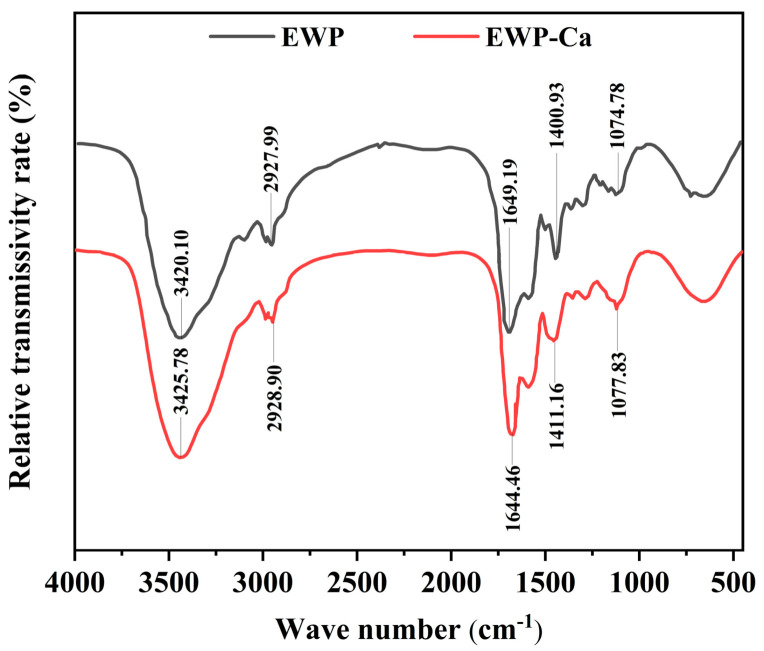
FTIR spectra of EWP and EWP-Ca from 4000 to 450 cm^−1^.

Many signals could be found in the FTIR spectrum. As usual, the first part was probably caused by strong O-H and N-H stretching vibrations, where the broadband is due to hydrogen-bonded O-H groups [34]. As shown in Figure 3, after chelating with calcium, the peak intensity at 3420.10 cm^−1^ was moved to 3425.78 cm^−1^. The change in the characteristic peak may be due to the formation of coordination bonds between nitrogen atoms and calcium ions by offering their electron pairs [35]. Hydrogen bonds were replaced with N-Ca bonds. At the 1649.19 cm^−^^1^ peak, the EWP absorption band was characterized as an amide I band caused by the C=O stretching vibration. After calcium chelation, the band corresponding to the amide I group was reduced to 1644.46 cm^−^^1^, indicating that the C=O group was involved in binding peptide and calcium. Meanwhile, the peak intensity at 1400.93 cm^−^^1^, characterized as the absorption peak of -COO-, moved to a higher peak of 1411.16 cm^−^^1^ after being chelated with calcium, indicating that COO- may be combined with Ca^2+^ and transformed into -COO-Ca. Furthermore, the peak at 1074.78 cm^−^^1^ of EWP may be due to the symmetrical stretching vibration of –PO_3_. It moved to a higher peak of 1077.83 cm^−^^1^ after being chelated with calcium, indicating that the phosphate groups may also be involved in calcium binding.

FTIR spectroscopy was usually used to report the chemical structures of the samples, particularly in the interaction of metal ions and organic ligand groups in peptides [29,36]. The FTIR results in this experiment reflect that the calcium-binding sites on EWP are mainly carboxyl groups, phosphate groups, and amino groups, and peptide bonds may also participate in peptide–calcium binding. As known, every substance has its FTIR spectrum. However, the peak position of basic chemical groups is roughly similar [12]. The data in our experiment were similar to other sources for calcium-binding peptides [18,37,38]. In addition, the amino acid composition of EWP and EWP-Ca was analyzed in our previous research [39]. The results showed that aspartic acid (Asp), glutamic acid (Glu), and cysteine (Cys) in EWP might bind calcium and thus form the EWP-Ca complex. Acidic amino acids in peptide sequences can provide carboxyl groups that are the main calcium-binding sites. Cys contributing to calcium binding may be attributed to the sulfhydryl group of its side chain.

### 3.3. Raman Spectrum Analysis

**Figure 4:**
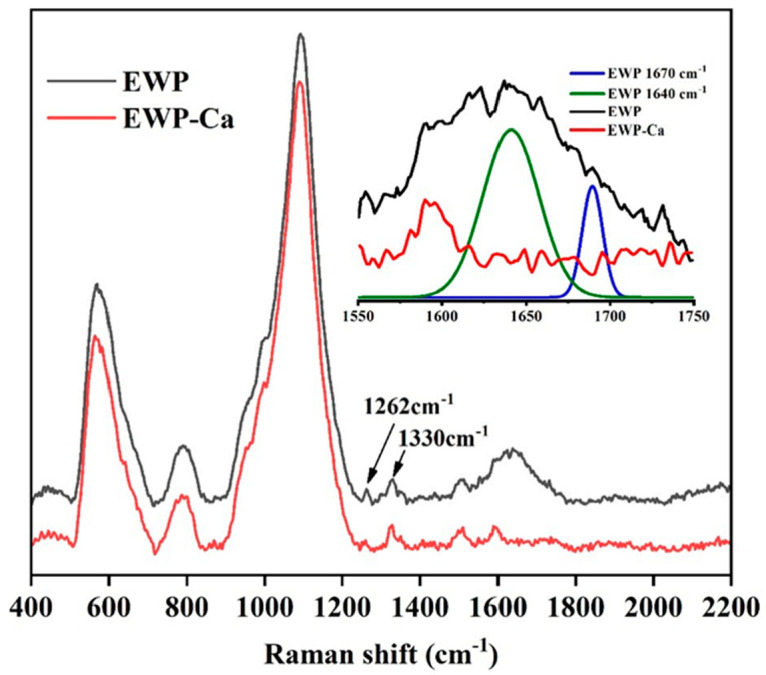
Raman spectra of EWP and EWP-Ca from 600 to 2200 cm^−1^.

Information about the structure and conformation of biomolecular can be obtained through Raman spectrum analysis [40]. As shown in Figure 4, the Raman spectral peak at the position of 1640 cm^−^^1^ is mainly caused by the stretching vibration of C=O, and the peak at the position of 1330 cm^−^^1^ corresponds to the symmetrical stretching vibration of the COO- of the amino acid side chain [25]; When the egg white peptide was binding with calcium, the peak intensities of C=O and COO- were decreased. Therefore, it can be concluded that carboxyl and carbonyl groups were the primary binding sites between EWP and calcium. The FTIR results further confirmed these findings. In addition, the peak at 1262 cm^−^^1^ represents α-helix, and the peak at 1670 cm^−^^1^ represents β-sheet [41]. It can be found that chelating with calcium caused the reduced peaks, which indicated the decrease in α-helix and β-sheet.

### 3.4. Promote Calcium Absorption Analysis of EWP-Ca

**Figure 5:**
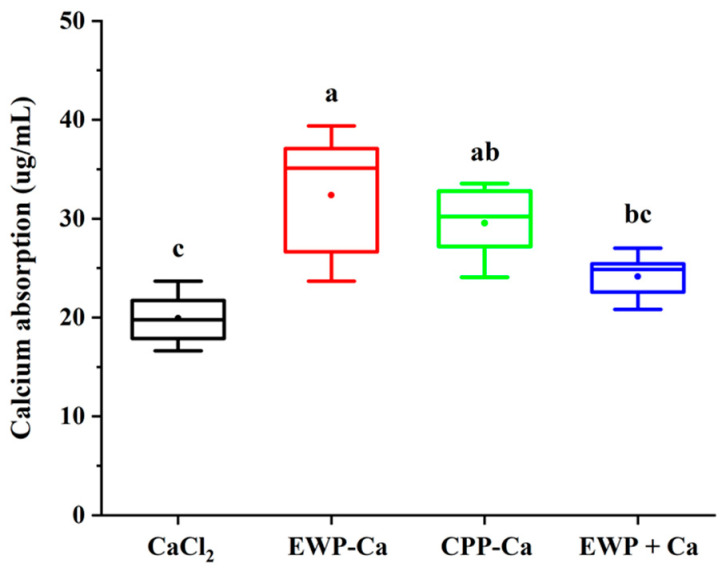
The calcium absorption of CaCl_2_, EWP-Ca, CPP-Ca, and EWP + Ca. The different letters indicate a significant difference (*p* < 0.05).

The intestinal everted sac model in rats was used to simulate the intestinal environment in vivo and evaluate the absorption of active substances. As a popular calcium supplement product of protein peptide–calcium product on the market, CPP–Ca was used as the control group together with CaCl_2_ in this experiment. As shown in Figure 5, calcium absorption of EWP-Ca (32.38 ± 6.83 μg/mL) was significantly higher than CaCl_2_ (19.93 ± 2.84 μg/mL) (*p* < 0.05), while higher than the EWP + Ca (24.14 ± 2.43 μg/mL) (*p* < 0.05), although it had no significant difference with CPP-Ca (29.56 ± 3.94 μg/mL) (*p* > 0.05). This revealed that the EWP-Ca and CPP-Ca had similar effects which could promote the absorption of calcium.

### 3.5. Nano LC-ESI-MS/MS Analysis

A high-resolution mass spectrometer was used to measure EWPs which could chelate calcium. As shown in Table 1, 39 peptides were found in egg white peptides, with the main component molecular weight distribution between 1000 and 2000 Da. It is worth noting that acidic amino acids including Asp and Glu in EWP-Ca can be observed in these peptides. A comparison of the Food Composition Databases with our previous studies about amino acid composition of egg white protein fraction [42] and EWP-Ca [39] revealed a significant increase in the proportion of acidic amino acids. This is consistent with a previous study that the acidic amino acids of Glu and Asp are beneficial for calcium ions chelating with peptides [15]. Liu et al. believe calcium-binding peptide possesses essential residues: Glu, Arg, Asp, Gly, and hydrophobic amino acid content, which play an important role in calcium-binding capacity [18]. In the peptides sequence results, Glu, Arg, and Asp are frequent, and there are 22, 14, and 34 peptides containing the three amino acids, respectively. These results indicate that EWP is an ideal source for preparing peptides–calcium chelate.

### 3.6. Stability of EWP-Ca during the Sterilization Process

#### 3.6.1. Fluorescence Spectra and CD Spectra

**Figure 6:**
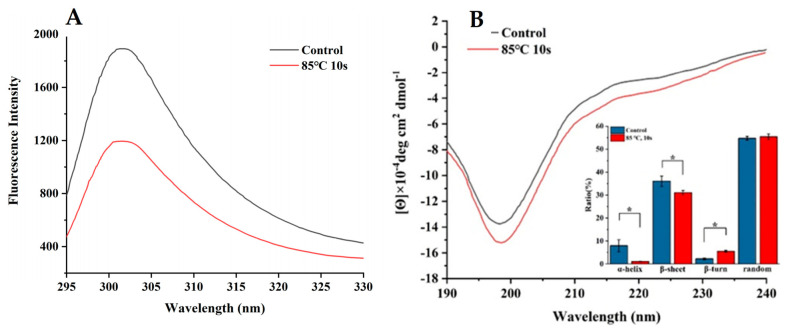
Fluorescence spectra (**A**) and circular dichroism spectra (**B**) of EWP-Ca and sterilization treatment EWP-Ca. * indicates significant differences (*p* < 0.05).

The tryptophan residue at 295 nm excitation produces fluorescence intensity at a wavelength within the range of 300–400 nm, with the potential energy of tryptophan fluorescence bands indicating a change in position of the tryptophan residue in the protein and changes in the protein structure [43]. As shown in Figure 6A, after sterilization, the endogenous fluorescence of EWP-Ca decreased significantly. It is likely that the sterilization treatment caused the structure of EWP-Ca to be folded or form aggregates with tryptophan buried far from the water interface, resulting in a decrease in its fluorescence intensity.

CD spectroscopy is typically used to measure the secondary structures of proteins and peptides, which is an established and valuable technology [44,45]. Figure 6B shows the CD spectra of EWP-Ca before and after sterilization. The above study found that egg white peptides were mainly oligopeptides composed of ten amino acid residues, and these oligopeptides could form secondary structures through self-assembly. After the sterilization process, the secondary structure of the egg white peptide changed obviously, and the ordered structure (α-helix) decreased significantly. The increase in β-turn could indicate that the peptide was desaturated by heat and had re-folding or agglomeration changes, which was consistent with the decrease in fluorescence intensity in the above study. During the formation of egg white peptide-chelated calcium, the hydrogen bond, hydrophobic interaction and electrostatic interaction between Ca^2+^ and egg white peptide cause the egg white peptide to maintain a specific spatial conformation [45] cause the egg white peptide to maintain a specific spatial conformation. Fluorescence spectrum and circular results show that sterilization does change the structure of egg white peptides and that it will change the interactions between Ca^2+^ and egg white peptides. Thus, the thermal stability of egg white peptide calcium chelate was affected.

#### 3.6.2. Calcium-Binding Capacity Analysis

As described in Table 2, the calcium-binding capacity of EWP-Ca at room temperature was 65.25%. Wang et al. reported that a novel calcium-binding peptide isolated from wheat germ protein hydrolysates was purified and confirmed to be FVDVT (Phe-Val-Asp-Val-Thr), and the calcium-binding capacity of FVDVT reached 89.94 ± 0.75% [46]. Hou et al. investigated the optimal calcium-chelating condition of Antarctic krill peptides, and the calcium-chelating capacity can reach 0.046 mmol/g when set as the optimal condition [47]. Guo et al. fractionated and identified the Alaska pollock skin collagen-derived mineral chelating peptides, and the results showed that calcium chelating activity was 11.52 ± 2.23 nmol/µmol [48]. The difference in the calcium-binding capacity of different kinds of peptides is probably due to the molecular weight and acidic amino acid content of peptides. The peptides with low molecular weight could favor calcium-binding peptides [26]. In addition, several peptides rich in acidic amino acids have been demonstrated to exert positive contributions for calcium chelation [49,50].

After sterilization, the calcium-binding capacity of EWP-Ca significantly decreased to 41.19% (*p* < 0.05). It can be preliminarily inferred that EWP-Ca was unstable under high-temperature conditions. High temperatures destroyed the structure of the peptide, resulting in the destruction of the peptide–calcium binding bond and the release of calcium. However, high-temperature treatment did not wholly dissociate calcium ions, indicating that the bond between calcium and peptide has a certain tolerance to high temperature.

## 4. Conclusions

In conclusion, we found that EWP-Ca can effectively improve the absorption of calcium by the intestinal everted sac model, and that the absorption is similar to the CPP–Ca. In addition, egg whites are easy to obtain and the price is low, which makes EWP-Ca, an attractive new type of calcium supplement. The peptides obtained in related studies are mostly oligopeptides, consisting of 5–8 amino acid residues. However, the egg white peptides in this study are limited by the preparation process. The peptides obtained are mostly composed of about 10+ amino acid residues, making the peptide conformational easily affected by environmental factors, thereby decreasing the interaction between calcium ions and peptides. This may be the reason why the sterilization treatment can influence calcium-binding capacity. Subsequent research will be based on the perspective of industrial development, and it is possible to use a complex enzyme hydrolysis method to improve hydrolysis efficiency, reducing the influence of peptide conformational changes on the calcium binding. Future studies could also investigate how processes such as microencapsulation and Pickering emulsification immobilize the EWP-Ca conformation and improve thermal stability.

## Figures and Tables

**Table 1:** The sequence description of EWP-Ca peptides.

No.	Peptide Sequence	Molecular Weight (Da)
1	FAGDD	523.19
2	NFGPKG	618.31
3	GFGFVTF	773.37
4	FAGDDAPR	847.38
5	SYELPDGQV	1006.46
6	ADLIAYLKK	1033.62
7	ALESPERPF	1044.52
8	FLFDKPVSPL	1161.64
9	KLDKENAIDRA	1271.68
10	SLGTADVHFERK	1358.69
11	RGDLGIEIPAEKV	1395.77
12	GDLGIEIPAEKV	1395.77
13	KLEEAEKAADESE	1447.67
14	VEPEILPDGDHDL	1447.68
15	ARFEELNADLFR	1479.75
16	FVGGIKEDTEEHH	1496.69
17	HLEINPDHPIVET	1512.76
18	NDLFENTNHTQVQ	1558.7
19	DGFIDKEDLHDML	1562.69
20	HQGVMVGMGQKDSY	1567.68
21	VEPEILPDGDHDLK	1575.78
22	VGGIKEDTEEHHLR	1618.81
23	VVSSIEQKTEGAEKK	1631.87
24	KLEEAEKAADESERG	1660.79
25	QKLEEAEKAADESE	1731.83
26	DVSNADRLGFSEVELV	1748.86
27	EKNPLPSKETIEQEK	1768.92
28	GIITNWDDMEKIWH	1772.82
29	DIFEANDLFENTNHT	1778.77
30	IQLVEEELDRAQERL	1839.97
31	KLEEAEKAADESER	1844.91
32	QLIDDHFLFDKPVSPL	1882.98
33	RIQLVEEELDRAQER	1882.99
34	WIDNPTVDDRGVGQAAIR	1982
35	DIFEANDLFENTNHTQV	2005.9
36	DIFEANDLFENTNHTQVQ	2133.96
37	AVAGNISDPGLQKSFLDSGYR	2194.1
38	DDHDPVDKIVLQKYHTINGH	2343.16
39	GFGFVTFDDHDPVDKIVLQKY	2439.21

**Table 2:** The calcium-binding capacity of EWP-Ca and sterilization treatment EWP-Ca.

	Room Temperature	85 °C 10 s
Calcium-binding capacity (%)	65.25 ± 2.73 ^a^	41.19 ± 3.48 ^b^

Values are given as the mean ± SD. Different letters (^a^ and ^b^) within a row indicate significant differences (*p* < 0.05).

## Data Availability

Data are available upon request.
